# Adenoviral Vectors Stimulate Glucagon Transcription in Human Mesenchymal Stem Cells Expressing Pancreatic Transcription Factors

**DOI:** 10.1371/journal.pone.0048093

**Published:** 2012-10-26

**Authors:** Arnaud Zaldumbide, Françoise Carlotti, Manuel A. Gonçalves, Shoshan Knaän-Shanzer, Steve J. Cramer, Bart O. Roep, Emmanuel J. H. J. Wiertz, Rob C. Hoeben

**Affiliations:** 1 Department of Molecular Cell Biology, Leiden University Medical Center, Leiden, The Netherlands; 2 Department of Nephrology, Leiden University Medical Center, Leiden, The Netherlands; 3 Department of Immunohematology and Blood Transfusion, Leiden University Medical Center, Leiden, The Netherlands; 4 Department of Medical Microbiology, University Medical Center Utrecht, Utrecht, The Netherlands; University of Pittsburgh, United States of America

## Abstract

Viral gene carriers are being widely used as gene transfer systems in (trans)differentiation and reprogramming strategies. Forced expression of key regulators of pancreatic differentiation in stem cells, liver cells, pancreatic duct cells, or cells from the exocrine pancreas, can lead to the initiation of endocrine pancreatic differentiation. While several viral vector systems have been employed in such studies, the results reported with adenovirus vectors have been the most promising *in vitro* and *in vivo*. In this study, we examined whether the viral vector system itself could impact the differentiation capacity of human bone-marrow derived mesenchymal stem cells (hMSCs) toward the endocrine lineage. Lentivirus-mediated expression of Pdx-1, Ngn-3, and Maf-A alone or in combination does not lead to robust expression of any of the endocrine hormones (i.e. insulin, glucagon and somatostatin) in hMSCs. Remarkably, subsequent transduction of these genetically modified cells with an irrelevant early region 1 (E1)-deleted adenoviral vector potentiates the differentiation stimulus and promotes glucagon gene expression in hMSCs by affecting the chromatin structure. This adenovirus stimulation was observed upon infection with an E1-deleted adenovirus vector, but not after exposure to helper-dependent adenovirus vectors, pointing at the involvement of genes retained in the E1-deleted adenovirus vector in this phenomenon. Lentivirus mediated expression of the adenovirus E4-ORF3 mimics the adenovirus effect. From these data we conclude that E1-deleted adenoviral vectors are not inert gene-transfer vectors and contribute to the modulation of the cellular differentiation pathways.

## Introduction

The use of adult stem cells in regenerative medicine procedures is appealing. In this respect the use of human mesenchymal stem cells (hMSCs) is particularly attractive since these cells can be isolated with relative ease from living donors. MSC cultures can be expanded *ex vivo* and the cultured cells can be enticed to differentiate in ectodermal, mesodermal and endodermal cell types, albeit with varying efficiencies.

A tissue that warrants particular interest in regenerative medicine is the endocrine pancreas. The endocrine pancreas contains the Islets of Langerhans. These islets consist of cell clusters, which contain the major pancreatic hormone-producing cells, such as the α-cells, producing glucagon, the β-cells, which synthesize insulin, the δ-cells, producing somatostatin, and the PP cells that generate pancreatic polypeptide. These hormones are involved in, among others, the regulation of glucose homeostasis. In diabetes type 1 the glucose homeostasis is disturbed as a result of the immune-mediated destruction of the insulin-producing cells.

While whole pancreas transplantation and, more recently, islet transplantation show promising results for type 1 diabetes, the shortage of organ donors warrants exploration of new therapeutic avenues. The generation of islet cells from adult stem cells offers the prospect of a permanent cure independently of organ donations. However, so far no robust protocols have been developed for generating insulin-producing cells from adult human stem cells. A limited number of studies demonstrated the feasibility of *in vitro* derivation of insulin-producing cells from hMSCs using a combination of defined media or overexpression of key regulators of pancreatic development [1]–[6].

In an alternative approach, several groups have explored the option of transdifferentiation into insulin-producing cells of differentiated cells from embryologically related organs such as liver and pancreatic exocrine tissue. In many of these studies, viral vectors have been used to force the expression of key differentiation factors including Pdx-1, Pax-4, Maf-A, Ngn-3, NeuroD, and Betacellulin. Ferber and co-workers showed that ectopic overexpression of Pdx-1 in the liver with a first-generation, i.e. early-region 1 (E1)-deleted, adenovirus type 5 (HAdV-5) vector resulted in formation of insulin-producing cells. This procedure decreased hyperglycemia in streptozotocin-induced diabetes in mice [7]. Similar liver transdifferentiation was obtained using the adenovirus-mediated transfer of NeuroD and Betacellulin [8]. In contrast, Wang and collaborators were not successful when they employed AAV-mediated gene transfer of Pdx-1 and Ngn-3. Hyperglycemia was reverted in diabetic mice only when AAV-mediated transfer of Pdx-1 and Ngn-3 was combined with co-administration of an irrelevant adenoviral vector [9]. *In vitro*, human adult liver cells could be efficiently transdifferentiated into functional β-cells after adenovirus-mediated ectopic expression of Pdx-1 [10]. Also, adult human pancreatic duct cells were converted into insulin-expressing cells using the adenovirus-mediated expression of Ngn-3 or NeuroD/Beta2 [11]. Finally, it was shown that adenoviral vector-mediated co-expression of Pdx-1, Ngn-3, and Maf-A in the exocrine portion of the pancreas restored β-cell function and ameliorated hyperglycemia in adult mice [12].

Here we report on our studies that aimed at forcing differentiation of bone marrow-derived hMSCs by ectopic expression of the differentiation factors Pdx-1, Ngn-3, and Maf-A using lentiviral vector-mediated gene transfer. While hMSCs could be transduced very efficiently, without apparent signs of vector-associated cytotoxicity, we were unable to achieve robust differentiation of these cells toward the endocrine pancreatic lineage. Remarkably, we observed that subsequent transduction with an adenoviral vector carrying an irrelevant transgene stimulates glucagon gene expression, but neither induces insulin nor somatostatin gene expression. We provide evidence that this glucagon-specific stimulatory effect resulting from adenoviral vector infection is attributable to the expression of adenovirus genes retained in the viral vector backbone. Moreover, we demonstrate that lentiviral vector expression of the adenovirus E4-ORF3 protein (UniProt ID P04489) partially mimics the adenoviral vector-induced up-regulation of glucagon gene expression. Of note, the adenoviral vector transduction was accompanied by changes in histone H4 acetylation. These observations, together with the fact that the adenovirus E4 region is expressed upon E1-deleted adenovirus vector infection, demonstrate that differentiation and transdifferentiation experiments that rely on first-generation adenoviral vectors should be interpreted cautiously as these gene carriers can contribute to the aspecific modulation of the cellular differentiation pathway(s) under investigation.

## Results

### Lentivirus-mediated Expression of Pdx-1, Ngn3 and Maf-A Fails to Initiate an Endocrine Differentiation Program in Human BM-MSC

**Figure 1:**
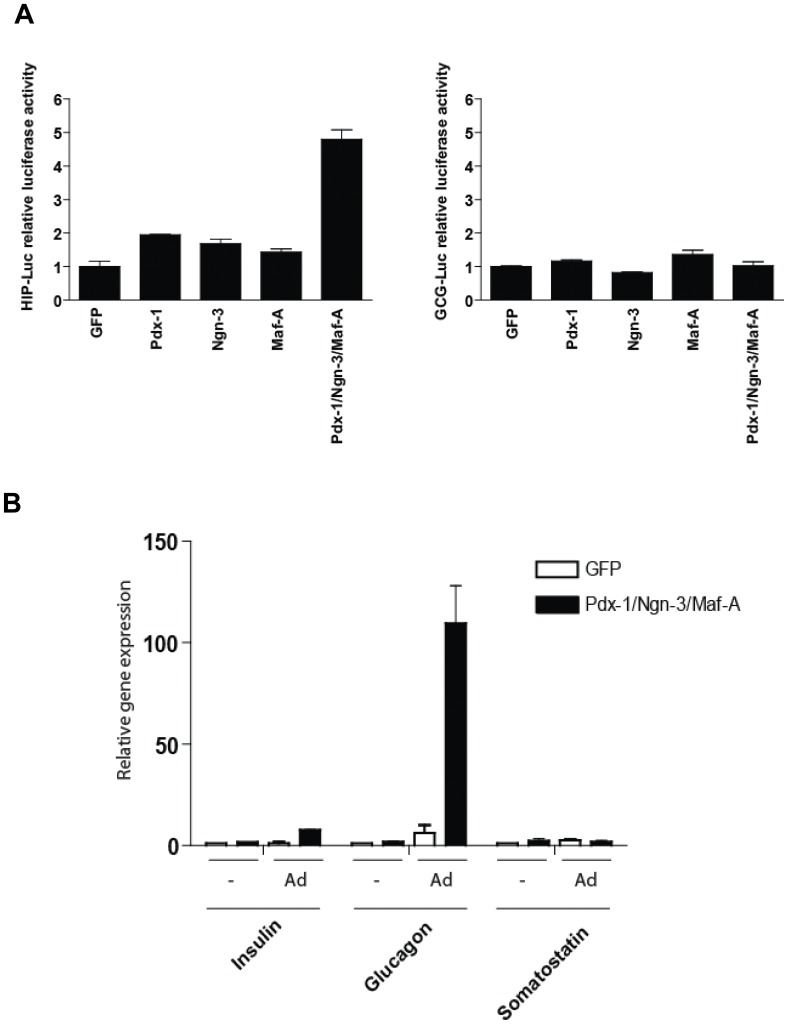
Pdx-1, Ngn-3, and Maf-A expression transactivate the human insulin promoter. (A) 293T cells transduced with the LV-HIP-Luc lentivirus (left panel) and with the LV-GCG-Luc lentivirus (right panel) were infected with with LV-CMV-Pdx-1 (MOI = 1); LV-CMV-Ngn-3 (MOI = 1); LV-CMV-Maf-A (MOI = 1) or with all tree lentiviruses combined at a total MOI of 1. Luciferase activity in cell lysates was analysed 48 h post-infection. Luciferase activity of the 293T-LV-HIP-Luc and 293T_LV-GCG-Luc exposed to the lentivirus LV-CMV-GFP was set to 1. (B) Exposure of BM-MSC cells overexpressing Pdx-1, Ngn-3, and Maf-A to an adenovirus carrying an irrelevant transgene specifically induces glucagon gene expression. Human BM-MSC were transduced with LV-CMV-GFP (MOI = 2) or LV-CMV-Pdx-1 (MOI = 2); LV-CMV-Ngn-3 (MOI = 2) and LV-CMV-Maf-A (MOI = 2). Four days post- transduction, the modified cells were maintained for 3 additional days before analysis or infected with HAdV-5/fib50-EF1α-DsRed (MOI = 30). Cells were analyzed for insulin, glucagon and somatostatin gene expression by qPCR 3 days post adenovirus infection (i.e. 1 week post lentivirus infection). Data are presented as mRNA level relative to GAPDH mRNA as a reference.

To test whether forced expression of key tissue-specific transcription factors could drive bone marrow-derived hMSC differentiation toward the endocrine lineage, we generated a set of lentiviral vectors containing cDNA for human Pdx-1, human Ngn-3 and human Maf-A. To assess the functionality of these vectors we studied their capacity to transactivate the human insulin gene promoter. To this end, we used 293T cells harboring a firefly luciferase gene driven by regulatory sequences from the human insulin promoter. As expected, exposing these 293T/hINS-luc cells to the combination of lentiviral vectors encoding Pdx-1, Ngn-3 and Maf-A, resulted in activation of the recombinant human insulin promoter. In contrast, expressing each of the three genes individually barely activated these heterologous insulin gene-derived sequences (Fig. 1A, left panel). Similar promoter transactivation studies conducted with the luciferase reporter construct driven by the glucagon promoter show that neither Pdx-1 alone nor combined with Ngn-3 and Maf-A activated the glucagon promoter (Fig. 1A, right panel).

To investigate whether the co-expression of Pdx-1, Ngn-3 and Maf-A in hMSCs could activate the endogenous islet-specific genes, we exposed hMSCs to these lentiviral vectors. However, this did not up-regulate expression of the insulin, glucagon or somatostatin genes (Fig. 1B). These results are unexpected in light of previous *in vivo* and *in vitro* studies, which utilized adenoviral vector-mediated transfer of these genes.

### E1-deleted Adenoviral Vector Infection Induces Glucagon Gene Expression in Human BM-MSC Expressing Endocrine Differentiation Factors

To assess whether different gene transfer vectors would affect the outcome of these directed differentiation experiments, hMSCs ectopically expressing Pdx-1, Ngn-3 and Maf-A were superinfected with an early region 1 (E1)-deleted adenoviral vector carrying an EF1α promoter-driven DsRed reporter gene (i.e. HAdV.EF1α.DsRed.F50). Since hMSCs do not express the Coxsackie-B virus and Adenovirus Receptor (CAR), which constitutes the primary attachment receptor for the human adenovirus type 5, HAdV.EF1α.DsRed.F50 was endowed with CAR-independent fiber derived from adenovirus type 50 [13]. Insulin, glucagon and somatostatin gene expression was evaluated. Remarkably, the ectopic expression of Pdx-1, Ngn-3 and Maf-A in hMSCs, combined with infection with HAdV.EF1α.DsRed.F50, increased glucagon gene expression up to 100-fold. This treatment had only minor effects on insulin and somatostatin gene expression (Fig. 1B).

**Figure 2:**
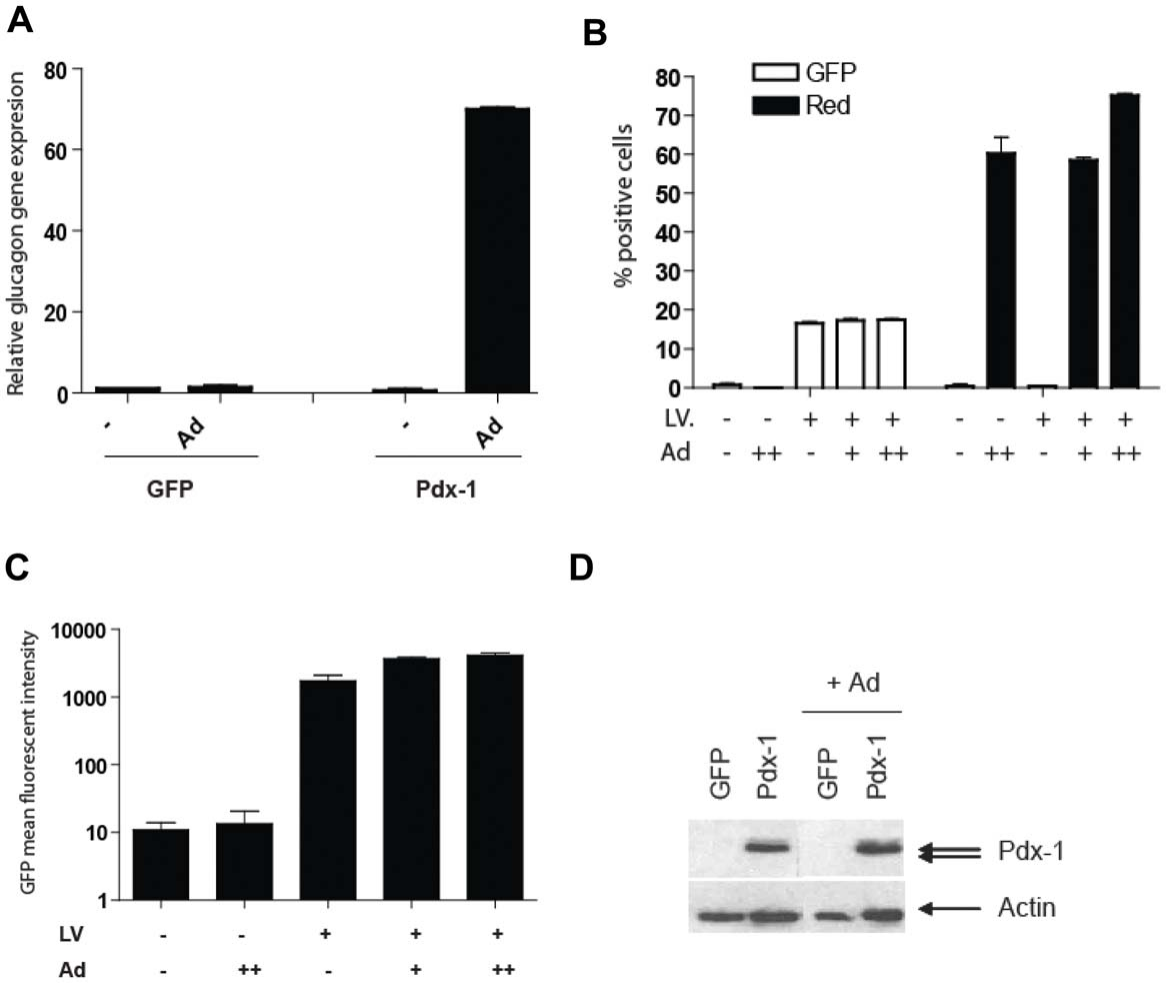
Ectopic Pdx-1 expression in combination with HAdV-5/fib50-EF1α-DsRed infection induces glucagon gene expression in bone marrow-derived MSC. (A) BM-MSC were transduced with LV-CMV-Pdx-1 (MOI = 2), and 4 days post-transduction the modified cells were exposed to HAdV-5/fib50-EF1α-DsRed at an MOI = 30. Glucagon gene expression was evaluated by qPCR and GAPDH was used as reference. (B–C) Adenovirus transduction does not stimulate CMV-promoter driven transgene. BM-MSC were transduced with LV-CMV-GFP and increasing amounts of HAdV-5/fib50-EF1α-DsRed virus (+: MOI = 10; ++: MOI = 30). Percentage of GFP and DsRed positive cells (B) and mean fluorescent intensity (C) were evaluated by FACS (Values are indicated +/− SD). (D) Western blot analysis of LV-CMV-Pdx-1 modified cells transduced with HAdV-5/fib50-EF1α-DsRed (MOI = 30). Pdx-1 expression level was evaluated by western blot using anti-Pdx-1 antibody; anti-actin was used as loading control.

Next, we evaluated the impact of adenoviral vector on hMSCs modified to express only Pdx-1. Similarly, lentiviral vector-mediated ectopic expression of Pdx-1 in hMSCs had no effect on the glucagon mRNA level whereas subsequent transduction of these cells with HAdV.EF1α.DsRed.F50 induced glucagon gene expression by approximately 80-fold (Fig. 2A). Importantly, adenoviral vector infection did not stimulate glucagon gene expression in hMSCs modified to express GFP, demonstrating that the induction of glucagon gene expression is dependent on both adenoviral vector transduction and forced expression of Pdx-1.

### E1-deleted Adenoviral Vector Transduction does not Transcriptionally Activate a CMV-promoter Driven Transgene

A trivial explanation for these unexpected results could be that the adenoviral vector transduction process, or the residual viral gene expression from the vector backbone, enhances the CMV promoter used to drive expression of the transcription factor genes. To test this hypothesis, hMSCs were transduced with LV-CMV-GFP at a relatively low multiplicity of infection (MOI) of 0.5 infectious units per cell. This resulted in approximately 20% GFP-positive cells. These cultures were transduced with HAdV.EF1α.DsRed.F50 or were mock-infected after which the GFP and DsRed expression parameters were monitored by flow cytometry. Although the HAdV.EF1α.DsRed.F50 vector was able to efficiently transduce the CAR-negative hMSCs (increasing vector doses led to up to 80% DsRed-positive cells) without any sign of toxicity (Fig. 2B), the GFP mean fluorescent intensity was not affected by adenoviral vector transduction (Fig. 2C). Similarly, adenoviral vector transduction of Pdx-1-expressing hMSCs did not alter Pdx-1 protein levels (Fig. 2D). Taken together, these data suggest that the adenoviral vector effect on glucagon expression was not the result of an increase of Pdx-1 expression due to transcriptional activation of the CMV promoter.

### E1-deleted Adenoviral Vector Transduction Leads to Epigenetic Changes in Human BM-MSC

**Figure 3:**
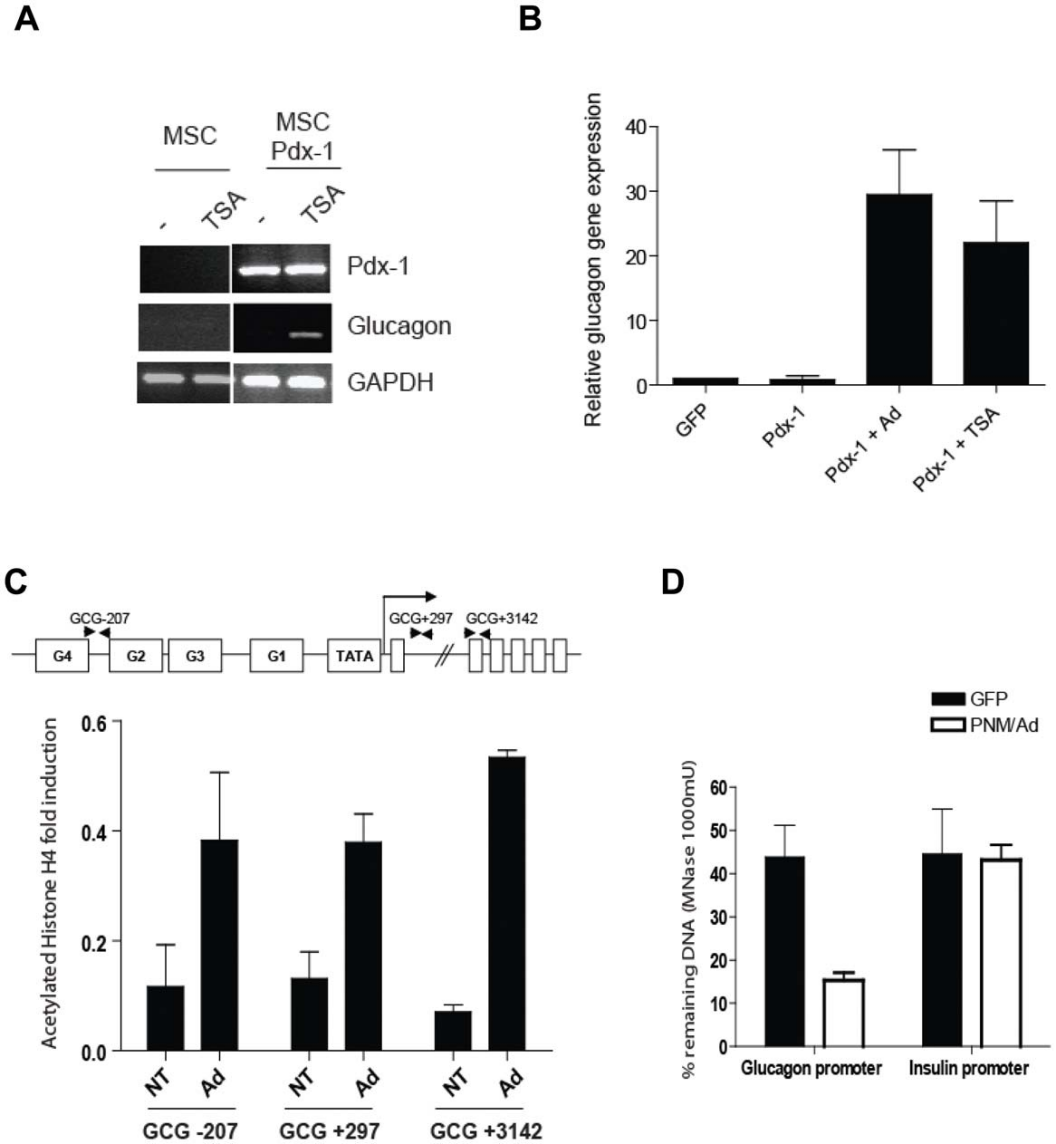
E1-deleted adenoviral vector transduction triggers epigenetic changes in BM-MSC. (A–B) LV-CMV-GFP or LV-CMV-Pdx-1-modified BM-MSC were treated for 3 days with TSA (1 µM). Pdx-1 and glucagon gene expression was assessed by RT-PCR (A) or qPCR (B). GAPDH was used as reference. (C) ChIP analysis of acetylated histone H4 in the human glucagon gene in BM-MSC modified by adenoviral vector (Ad) or in control non-transduced cells (NT). Position of the primer sets over the glucagon gene are indicated: GCG-207 = glucagon promoter; GCG+297 = Intron 1; GCG+3142 Exon 2. (D) Micrococcal accessibility assay of the glucagon promoter upon adenoviral transduction. DNA from BM-MSC (GFP) or adenovirus-modified BM-MSC (Pdx-1/Ngn-3/Maf-A) was extracted and digested with 1000 mU of micrococcal nuclease. Digested DNA was compared to undigested DNA by qPCR (set to 100%).

Chromatin structure is a major determinant in transcriptional regulation. Therefore we evaluated whether adenoviral vector transduction could trigger epigenetic changes in hMSCs. To this end, hMSCs were transduced with LV-CMV-Pdx-1, and 4 days post-transduction the histone deacetylase inhibitor trichostatin A (TSA) was added to the cultures, after which the cells were cultured for an additional 3-day period. In presence of TSA, Pdx-1 expression strongly induced glucagon expression to an extent similar to that resulting from adenoviral vector infection (Fig. 3A–3B). To test whether adenoviral vector transduction can alter the histone signature at the glucagon gene locus, a chromatin immunoprecipitation assay was performed on hMSCs before and after exposure to HAdV.EF1α.DsRed.F50. Using this set-up we assessed the histone H4 acetylation levels at the endogenous glucagon locus. Adenoviral vector transduction increased the histone H4 acetylation within the promoter (−207), intron 1 (+297), and exon 2 (+3142) (Fig. 3C). Consistent with an earlier study [14], lentiviral vector-mediated Pdx-1, Ngn-3 and MafA expression combined with adenovirus vector transduction led the glucagon promoter locus to become more sensitive to micrococcal nuclease digestion than the insulin gene (INS) (Fig. 3D).

### Expression of the Adenoviral E4-ORF3 Contributes to the Effect on Chromatin Changes

**Figure 4:**
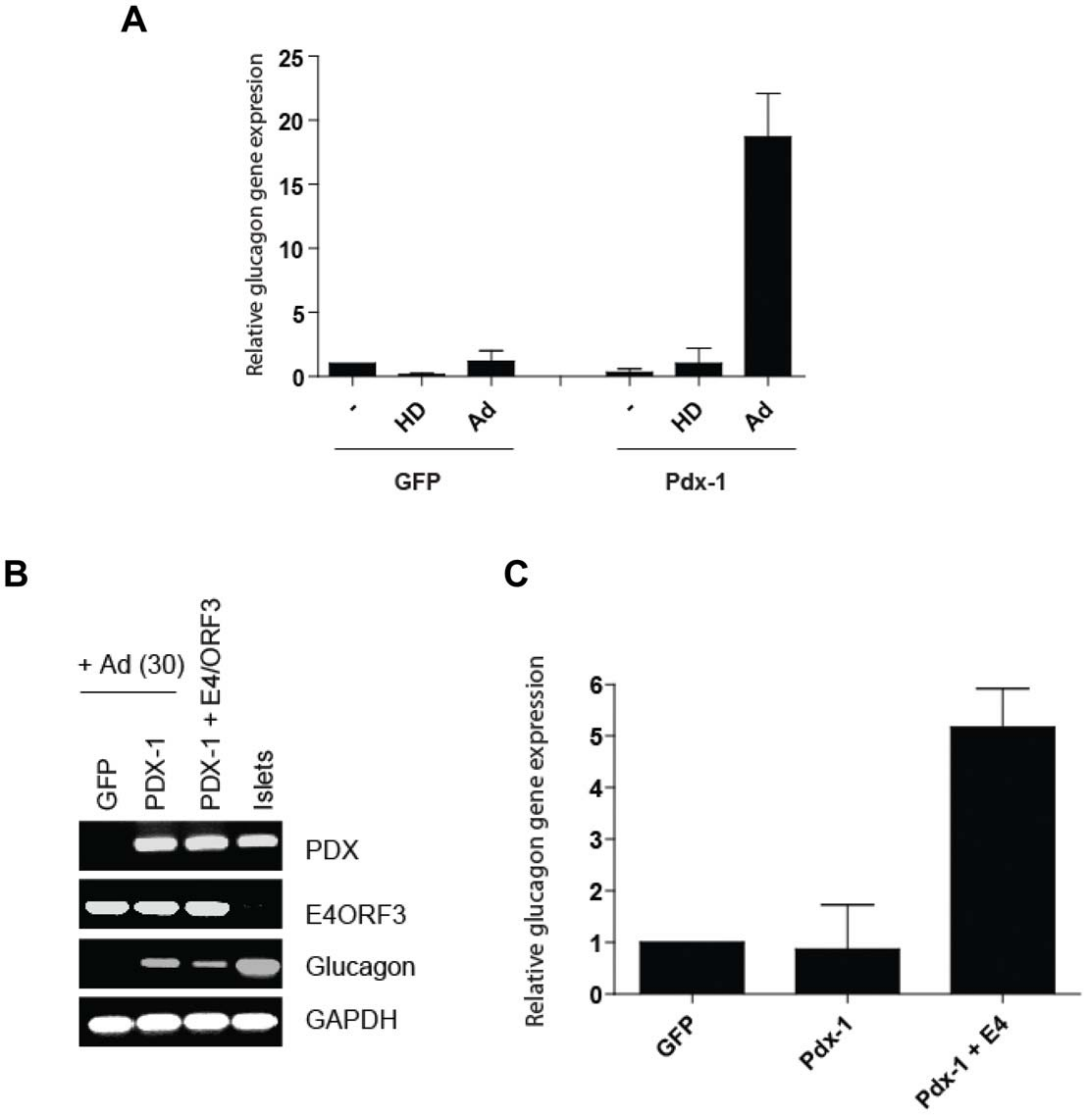
Genes retained in E1- deleted adenoviral vector affect glucagon gene expression. (A) LV-CMV-Pdx-1 modified cells were transduced with HAdV-5/fib50-EF1α-DsRed (MOI = 30) or HD-HAdV-5/Fib50. Four days post transduction glucagon gene expression was evaluated by qPCR. GAPDH was used as reference. (B) BM-MSC(GFP) or BM-MSC (Pdx-1) were transduced with HAdV-5/fib50-EF1α-DsRed or LV-CMV-E4-ORF3 (MOI = 2). Four days post transduction glucagon gene expression was evaluated by PCR (C) or qPCR (D). GAPDH was used as reference.

To determine whether the adenoviral vector effect on glucagon gene expression is attributed to the transduction by viral particles or to the residual expression of adenoviral genes, we compared glucagon gene expression upon transduction with viral gene-bearing HAdV.EF1α.DsRed.F50 and the helper-dependent adenoviral vector hcAd.FLPe.F50 [15]. The latter vector contains only the *cis*-acting adenovirus inverted terminal repeats and the packaging signal and thus does not deliver adenovirus protein-coding genes into target cells. As shown in Fig. 4A, no stimulation of glucagon gene expression was detected upon exposure of the Pdx-1-expressing hMSCs to the hcAd.FLPe.F50 vector, whereas transduction with the first-generation adenoviral vector increased glucagon gene expression approximately 20-fold. These data suggest that the adenovirus-specific effect on glucagon expression in Pdx-1 hMSCs can be attributed to the presence of adenovirus protein-coding genes in the adenovirus vector backbone.

Recently, the adenoviral E4-ORF3 gene product has been shown to induce heterochromatin formation thereby preventing p53 binding to its target genes [16]. To test whether E4-ORF3 could mimic the effect of first-generation adenoviral vector transduction in our cellular model system, we generated an *E4-ORF3*-containing lentiviral vector and assessed glucagon expression upon Pdx-1 and E4-ORF3 co-expression. Remarkably, 4 days after E4-ORF3-expressing lentiviral vector co-transduction, the amount of glucagon-specific transcripts was increased 5-fold as measured by RT-PCR (Fig. 4B) and q-RT-PCR (Fig. 4C). These data demonstrate that the E4-ORF3 gene product can either directly or indirectly synergize with Pdx-1 during the transcriptional activation process of the glucagon locus in hMSCs.

## Discussion

Bone marrow-derived hMSCs represent an attractive source of stem cells to be used in regenerative medicine since they are relatively easy to isolate (both autologous and allogeneic), purify, and expand *ex vivo*. The use of these primary adult stem cells is generally considered as safe. This has led to a large number of clinical studies involving administration of hMSCs. However, only few reports demonstrated robust differentiation of human MSC into insulin-producing cells [17]. Experiments performed in our laboratory employing lentiviral vectors expressing key master transcription factors to force differentiation of hMSC into insulin-producing cells did not yield an efficient protocol for the formation of endocrine-pancreatic cells. Indeed, overexpression of Pdx-1, Ngn3, Maf-A failed to induce a robust and reproducible differentiation of these cells *in vitro*. Phinney and colleagues reviewed the difficulties in achieving full differentiation of hMSCs by pinpointing the lack of standardized protocols for hMSC isolation and handling as a possible source of variation in the results obtained by various groups [18]. On the other hand, numerous *in vivo* and *in vitro* studies showed that expression of Pdx-1 promoted differentiation or transdifferentiation of various cell sources toward the endocrine pancreatic pathway. In an evaluation of the literature we noted that in most of these successful studies, adenoviral vectors had been used for gene transfer [3], [5]–[8], [10], [11]. This prompted us to evaluate specifically whether the particular choice of gene delivery vehicles could affect the differentiation capacity of adult human stem cells such as hMSCs.

Herein we show that the differentiation of bone marrow-derived hMSCs into α cell-like cells upon lentiviral vector-mediated expression of Pdx-1 was stimulated by exposure of the cells to a first-generation replication-incompetent adenoviral vector carrying a neutral transgene. Therefore it appears that a combined effect of ectopic Pdx-1 expression and adenoviral vector transduction was able to initiate an endocrine differentiation program. Similarly, the differentiation of human pancreatic islet derived-MSCs, which differentiate relatively poorly into adipocytes [19], was stimulated in presence of adenoviral vector particles (data not shown). These observations suggest that first-generation adenoviral vector transduction can potentiate differentiation stimuli. Previously, Wang et al. also highlighted the importance of the viral gene carrier in forced differentiation protocols by demonstrating that adenovirus transduction could enhance transdifferentiation of mouse liver cells into insulin-producing cells, whereas AAV-mediated Pdx-1 gene transfer could not [9]. The authors attributed this vector-specific effect to the induction of an interferon gamma response. This led them to suggest that the host immune response to the adenoviral vector is providing an environment that, in combination with the expression of the pancreatic transcription factors, facilitated transdifferentiation of hepatocytes into insulin-producing cells. This mechanism can be ruled out in our in vitro study.

Our data based on the use of the chromatin-remodeling drug TSA and epigenetic assays suggest that the adenoviral vector affects the chromatin structure of hMSCs increasing the histone H4 acetylation status and the accessibility of specific loci to transcription factors. The recurrent use of TrichostatinA or Sodium Butyrate treatment in differentiation protocol also support the idea of a crucial role of chromatin remodeling factor in the differentiation capacity of bone marrow mesenchymal stem cells [20], [21]. It is known that adenoviral transduction can result in massive changes in the gene expression pattern in the exposed cell population. Gene expression array studies demonstrated that the transcriptional activity of approximately 400 genes is altered at least by a factor 2 upon adenovirus infection [22]. A vast majority of these genes is implicated in cell cycle regulation, cell survival or apoptosis. The E1 region of adenovirus is the most involved in host gene disturbances and has previously been implicated in cell reprogramming [23]. Here we can certainly exclude the participation of E1 gene products since the adenoviral vector particles used are E1-deleted. Moreover, our viral vectors have been produced in PER.C6 cells to prevent contamination with E1-containing replication-competent adenovirus particles. We show that the adenoviral vector-dependent robust upregulation of glucagon expression in Pdx-1-positive hMSCs might be instead mediated by the adenovirus E4-ORF3 gene product since its overexpression partially mimics the adenovirus effect. E4-ORF3 is the most conserved protein among all adenovirus gene products. This 116 amino acid long multifunctional protein has been reported to be crucial for viral life cycle by promoting cell cycle-independent viral replication [24]–[27], regulating adenovirus mRNA splicing, and translation of late transcripts [28]. Although initially its function was suggested to overlap with the E4-ORF6 gene product, more recent data suggest that ORF3 and ORF6 have independent roles. E4-ORF3 is involved in PML nuclear bodies reorganization [25],[29]–[31]. Since nuclear bodies movement has been closely linked to chromatin accessibility, it is conceivable that E4-ORF3 is implicated in the chromatin modification seen upon E1-deleted adenoviral vector infection described in this study [32]. This hypothesis would be in apparent contradiction with the reported trimethylated H3K9 heterochromatin induction following E4-ORF3 expression in the U2OS tumor cell line. However, the authors clearly stated that the heterochromatin structure formed upon E4-ORF3 expression was highly selective for p53-targeted promoters [16]. Furthermore, the observation that Pdx-1 overexpression combined with either adenoviral vector transduction or TSA treatment stimulates glucagon transcription and histone H4 hyperacetylation at the glucagon locus, echoes earlier work on Pdx-1′s interaction with HDAC1 and HDAC2 and insulin promoter transactivation [33], [34]. This supports a mechanism by which E4-ORF3 interferes directly or indirectly with Pdx-1/HDACs interactions. This could lead to activation of Pdx-1 target genes and entices pancreatic differentiation leading to glucagon gene expression.

Surprisingly, no induction of insulin gene expression was observed in our system. Similarly, Wilson et al. reported that adenovirus-mediated overexpression of Pdx-1, Maf-A and NeuroD1 in islet derived-MSC had little effect on insulin gene expression, whereas it strongly induced glucagon gene expression [14]. Of note, in their study, the use of control adenovirus vector (expressing GFP only) already significantly increased the glucagon gene expression compared to untransduced cells. Importantly, this MSC-like cell population, which is likely to be derived from dedifferentiation [35], [36] of endocrine cells, is believed to retain an epigenetic memory making them more prone to differentiate toward the endocrine lineage. Furthermore, Mutskov and collaborators demonstrated that chromatin from bone marrow-derived hMSCs presented a high-level of H3 lysine 9 dimethylation, a hallmark of inactive genes, at the promoter of insulin locus [37]. Therefore it appears that the combined effect adenovirus vector and Pdx1 overexpression on chromatin opening is not sufficient to unlock the epigenetic silencing of the insulin gene.

Finally our data reveal that the potential effects of the viral gene-transfer vector should be taken into account if such vectors are used to instruct cellular differentiation programs or to delineate cellular signaling pathways. We show that conventional first-generation adenoviral vectors can have profound effects on the differentiation capacity of hMSCs. Such vectors have been successfully used for transdifferentiating liver, pancreatic duct, or exocrine tissue cells by overexpression of key transcription factors. Also adenovirus vectors are being developed as gene transfer vectors for expression of Oct4, Sox2, Klf4, and c-Myc for generation of induced pluripotent stem cells (iPS) [38], [39]. In conclusion, directed differentiation and transdifferentiation experiments that rely on first-generation adenoviral vectors should be interpreted cautiously as these gene carriers can contribute to the modulation of the cellular differentiation pathway(s) under investigation. As corollary, in these types of studies, the effects that the viral vector itself has on the cellular gene expression profile of the target cells need to be stringently controlled for.

## Materials and Methods

### Cells

293T cells were obtained from the American Type Culture Collection (Manassas, VA, USA). The human bone marrow was collected from anonymous surgical “waste” material in orthopedic surgery. In agreement with the pertinent Leiden University Medical Center guidelines, and in accordance with the Best Practices code of Dutch Federation of Biomedical Scientific Societies, and the based on article 467 of the ”Wet op de Geneeskundige Behandelingsovereenkomst (WGBO)” no informed consent is required for the use of anonymous and non-traceable body materials and the institutional ethic committee of the Leiden University Medical Center (LUMC; Leiden, the Netherlands) waived the need for patient consent. We confirm that based on the legislation the institutional ethic committee of the LUMC waived the need for approval for the use of surgical anonymous surgical “waste” material. hMSCs have been isolated as described previously [13] and characterized on their surface antigen expression profile by FACS analysis. The hMSC were confirmed to be positive for HLA-ABC, CD105, CD90, CD44, CD73, and lack expression of HLA-DR, CD34 and CD31 (Figure S1). Moreover, after 3 weeks under a lineage-specific differentiation protocol [31], these cells could efficiently differentiate into adipocytes and osteoblasts as seen respectively by Oil Red O and Alizarin Red S stainings (Figure S2). The hMSCs are efficiently amendable by lentiviral vector transduction using standard conditions (Figure S3).

### DNA Constructs

pRRL-CMV-Pdx-1-bcGFP, pRRL-CMV-Ngn-3-bcGFP and pRRL-CMV-Maf-A-bcGFP were generated by insertion of human Pdx-1 cDNA from pcDNA3.1-hPdx-1 (gift from Dr. Wendy Mac Farlane), human Ngn-3 cDNA from pAC-CMV-hNgn-3 (gift from Prof. Michael German) or Maf-A cDNA from pcDNA-hMaf-A-HSC (gift from Prof. Arun Sharma) into the lentiviral vector pRRL-CMV-bc-GFP [40].

cDNA encoding the E4ORF3 gene product was generated by RT-PCR from mRNA extracted from 1st generation adenoviral infected cells using the following primers Sal I/Xho I linker (Fw: GTCGACATGATTCGCTGCTTGAGGCTG; Rv: CTCGAGAGTTTCCAAAAGATTATCCAAAAC). The resulting fragment was then cloned into pRRL-CMV-bc-GFP [40] in order to generate pRRL-CMV-E4ORF3-bcGFP.

pRRL-hINS-Luc and pRRL-hGCG-Luc were generated by inserting the −326/+30 human insulin promoter or the −594/+136 human glucagon promoter upstream of the firefly luciferase reporter gene isolated from pGL3 (Promega).

### Vectors

Third generation self-inactivating lentiviral vectors containing the gene of interest, and the three helper plasmids (encoding HIV-1 gag/pol, HIV-1 rev, and VSV-G envelope glycoprotein) were co-transfected overnight into 293T cells using the calcium-phosphate DNA co-precipitation method. The vector supernatants were harvested after 48 and 72 hours post-transfection, passed through 0.45 µm pore-sized filters and stored at −80°C. The physical titers of the vector preparations were quantitated by antigen capture ELISA measuring HIV p24 levels (ZeptoMetrix Corporation, NY). This value has been converted to an infectious titer using the approximation that 1 ng of p24 equals 2500 infectious units. For transduction experiments, viral vector supernatants were added to fresh/regular culture medium supplemented with 8 µg/ml polybrene (Sigma), after which the cells were incubated overnight. The next day, the inoculum medium was replaced with fresh regular medium.

The HAdV.EF1a.DsRed.F50 was generated through homologous recombination in PER.C6 cells [41] transfected with 6.6 mg of Pac-treated pWE/Ad.AflII-rITR/Fib50 [42] and 1.4 mg of Pac-treated shuttle plasmid pAd.EF1a.DsRed. The latter construct contains a human EF1a-driven ORF encoding the red fluorescence protein DsRed plus sequences from the adenovirus type 5 DNA encompassing its “left-hand” portion and a genomic segment from nucleotide position 3511 through 6095. The lipofectamine (Invitrogen)-based transfection, as well as, the propagation, purification and titration of the vector stock was carried out essentially as described previously [43]. The generation and production of the helper-dependent hcAd.FLPe.F50 have been previously described [15], [43]. Titration of the hcAd.FLPe.F50 preparation was performed on HeLa cells containing the FLP-responsive construct pGS.pA+.DsRed [44].

### Protein Analyses

For determining the activity of the firefly luciferase reporter, cells were washed and treated with luciferase lysis buffer (25 mM Tris-Phosphate pH = 7.5, 2 mM CDTA, 2 mM DTT, 10% Glycerol, 1% Triton X-100). Luciferase activities were determined by luminometry using the Promega Luciferase Assay Reagent. Samples for Western analyses were prepared by boiling protein extracts with sample buffer (10% glycerol, 2% SDS, 60 mM Tris-Cl (pH 6.7), 2.5% beta-mercaptoethanol, and 2.5% bromophenol blue from a 1∶20-diluted saturated solution) for 5 minutes. Samples were analyzed on 10% polyacrylamide-SDS gels (30% acrylamide–bisacrylamide (37.5∶1); Bio-Rad) overlaid by a 5% stacking gel (30% acrylamide–bisacrylamide (37.5∶1); Bio-Rad). Proteins were transferred to Immobilon-P (Immobilon-P transfer membrane (polyvinylidene difluoride) Millipore, Etten-Leur, The Netherlands) and visualized using standard protocols with anti-Pdx-1 (Sigma) and anti-Actin (ICN Biomedicals, Inc., Zoetermeer, The Netherlands) as primary antibody. All antibodies were diluted in TBST (0.2% Tween 20, 150 mM NaCl, and 10 mM Tris) with 5% nonfat dried milk (Protifar Plus; Nutricia BV, Zoetermeer, The Netherlands).

### FACS Analysis

After gentle trypsinization hMSCs were resuspended in PBS supplemented with 0.1% BSA. Next, the samples were analyzed with a FACS LSRII flow cytometer (BD Pharmingen Inc., San Diego, CA). Using a forward scatter (FSC)/side scatter (SSC) representation of events, a region was defined in order to exclude cellular debris from the analysis. Percentages of GFP or DsRed-positive cells were determined in comparison to the negative control (non-transduced cells). Data analysis was performed using CellQuest 3.1 software (Becton Dickinson). For each sample, 10,000 events were collected.

For the characterization of hMSCs by cell surface antigen profiling, the cells were washed in PBS containing 0.1% BSA and subsequently incubated in 100 µl of PBS/0.1% BSA with the appropriate amount of antibody for 45 min at 4°C in the dark. Cells stained with the appropriate isotype control mouse IgG were used as negative control. The surface marker profiling of hMSCs was performed using the following antibodies: HLA-ABC-FITC, CD90-FITC, CD73-PE, CD73-PE, CD31-FITC, CD34-FITC, CD13-PE, CD44-PE, CD45-FITC, CD29-PE, CD49b-PE, CD49e-PE (BD Pharmingen Inc., San Diego, CA, USA), CD105-PE (eBioscience, Inc., San Diego, CA), HLA-DR-PE (CLB, Amsterdam, The Netherlands). Cells were then washed in PBS containing 0.1% BSA and analyzed as previously described.

### Reverse-transcription PCR and Real-time PCR

Total cellular RNA was extracted from tissues and cultured cells using Trizol and quantified by measuring OD260 using a Nanodrop 1000. Approximately 500 ng of RNA was reverse transcribed using Superscript RT II kit (Invitrogen, Karlsruhe, Germany). Expression of the genes of interest was detected using the following primers: Insulin Fw GCA GCC TTT GTG AAC CAA CA, Insulin Rv CGG GTC TTG GGT GTG TAG AAG; Glucagon Fw GAA TTC ATT GCT TGG CTG GTG AAA GGC, Glucagon Rv CAT TTC AAA CAT CCC ACG TGG CAT GCA; Somatostatin Fw CGT CAG TTT CTG CAG AAG TCC CTG GCT, Somatostatin Rv CCA TAG CCG GGT TTG AGT TAG CAG ATC, Pdx1 Fw CCC ATG GAT GAA GTC TAC C, Pdx1 Rv GTC CTC CTC CTT TTT CCA C; MafA Fw CAG TCC TGC CGC TTC AAG, MafA Rv ACA GGT CCC GCT CTT TGG; GAPDH Fw ACA GTC AGC CGC ATC TTC TT, GAPDH Rv AAT GAA GGG GTC ATT GAT GG. PCR amplifications were performed on a PTC-200 thermocycler (Biozym, The Netherlands). Real-time PCR runs were performed in triplicate using the SybrGreen master mix kit (Applied Biosystems) and an Applied Biosystems Step One Plus machine. Fold changes in gene expression were calculated using the 2^−ΔΔCt^ method with GAPDH transcripts serving as reference and LV-CMV-GFP modified cells set to 1.

### Chromatin Immunoprecipitation Assay

Formaldehyde cross-linking was performed for 30 minutes at room temperature. After washing, the cells were lysed at 4°C in ChIP buffer (0.75% SDS, 5% Triton X-100, 0.75 M NaCl, 5 mM EDTA, 2.5 mM EGTA, 100 mM HEPES pH = 7.6) supplemented with proteases inhibitors (Complete Mini, Roche). Samples were then sonicated using a Bioruptor water bath sonicator for 15 minutes (30 seconds on/30 seconds off). Immunoprecipitation was performed, overnight at 4°C, using an anti-acetylated histone H4 antibody (Upstate 06–866) or a rabbit IgG control on chromatin lysates in the presence of Protein A and Protein G beads. After washing the pulled-down fraction, the DNA was eluted from the beads with a ChIP elution buffer (1% SDS, 0.1 M NaHCO_3_).

Purified ChIP DNA from input and antibody-bound chromatin were analyzed by Real Time PCR as described previously using the following primers: hGCG−207(Fw) CTT AGTGATTTTCATGCGTGATTG; hGCG−207(Rv) TGGGA ATGGAGAGAGCAGCTT; hGCG+297(Fw) CACAGAGAGGAACTGAG ATGGAAA; hGCG+297(Rv) GCTTTGCGGCTTCGCTATAT; hGCG+3142(Fw) GACACAGAGGAGAAATCCAGGTATTAA; hGCG+3142(Rv) CAGGCTTTATTCCAACCATATTGA; hGAPDH(prom)(Fw): TCCCAAAGTCCTCCTGTTTCA; hGAPDH(prom)(Rv): CAGCAGGACACTAGGGAGTCAA.

Quantification was performed as described previously [45] using the formula [(IP/In)_αH4_^T^−(IP/In)_IgG_^T^]/[(IP/In)_αH4_^C^−(IP/In)_IgG_^C^] where IP/In = 2^−(Ct(IP)−Ct(In))^ and T = Target sequence; C = GAPDH promoter sequence.

### Micrococcal Nuclease Digestion Assay

The assay was conducted as described previously [14]. DNA from approximately 5×10^5^ cells was digested with 1000 milliunits of micrococcal nuclease (Sigma-Aldrich, St. Louis, MO) for 5 minutes at room temperature. Subsequently, the resulting DNA was analyzed by qPCR targeting insulin (hINS) or glucagon (hGCG) promoters using the primer/probe sets hINS −275 and hGCG −207 described above. Quantification was performed as follow: % remaining DNA = [2^−(Ct1000−Ct0)^]×100.

## Supporting Information

Figure S1 **Human MSC characterization.** Surface antigen profiling of hMSC (light grey). A mouse IgG isotype control was used as negative control (dark grey).
(TIF)

Figure S2 **Differentiation capacities of hMSC in osteoblasts and adipocytes.** After 3 weeks differentiation of hMSC, lipid droplets characteristic for adipocytes (upper panel) and calcium deposit characteristic for osteoblast (lower panel) were visualized respectively by Oil Red O or Alizarin Red S staining.
(TIF)

Figure S3 **Transduction efficiency of hMSC by lentiviral vector was assessed with LV-CMV-GFP (MOI = 2).** GFP expression was determined by microscopy and FACS. Untransduced MSC were used as negative (light grey) (C).
(TIF)
