# Supplementary figures and images for: Adenoviral Vectors Stimulate Glucagon Transcription in Human Mesenchymal Stem Cells Expressing Pancreatic Transcription Factors
Source: PLoS One. 2012 Oct 26;7(10):e48093. doi: 10.1371/journal.pone.0048093 (PMC3482184; doi:10.1371/journal.pone.0048093)

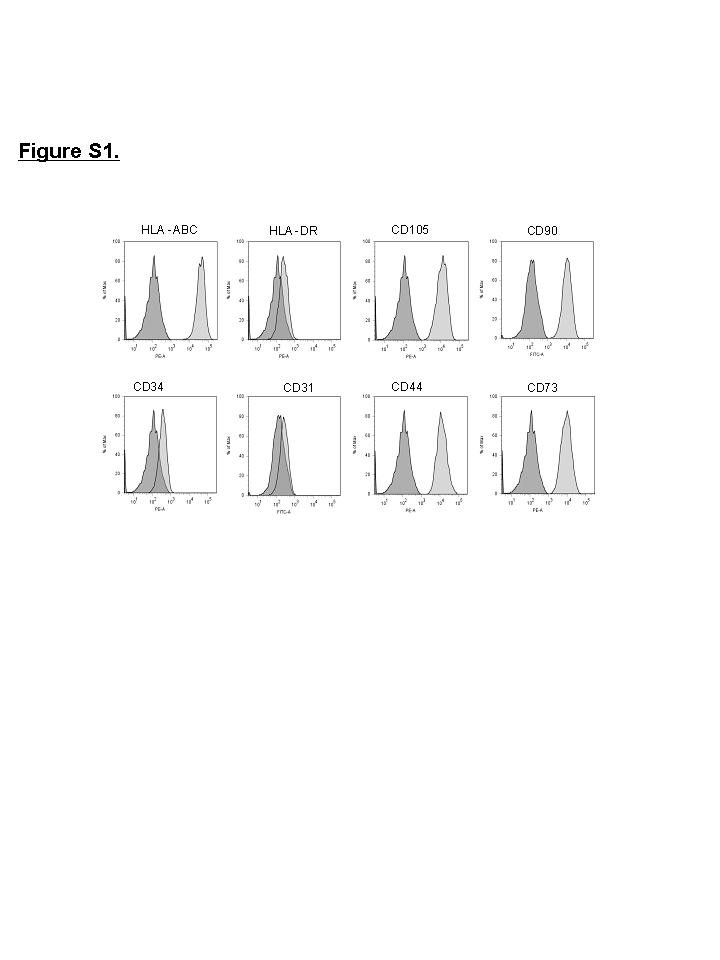

Supplement: Figure S1 — Human MSC characterization. Surface antigen profiling of hMSC (light grey). A mouse IgG isotype control was used as negative control (dark grey). (TIF) [file pone.0048093.s001.tif]

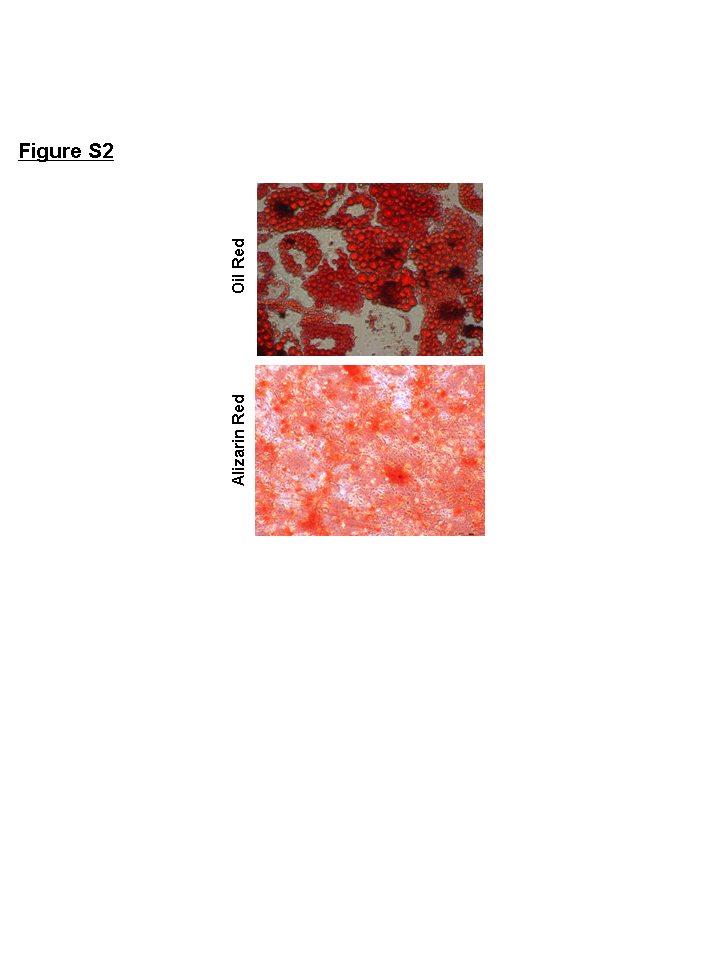

Supplement: Figure S2 — Differentiation capacities of hMSC in osteoblasts and adipocytes. After 3 weeks differentiation of hMSC, lipid droplets characteristic for adipocytes (upper panel) and calcium deposit characteristic for osteoblast (lower panel) were visualized respectively by Oil Red O or Alizarin Red S staining. (TIF) [file pone.0048093.s002.tif]

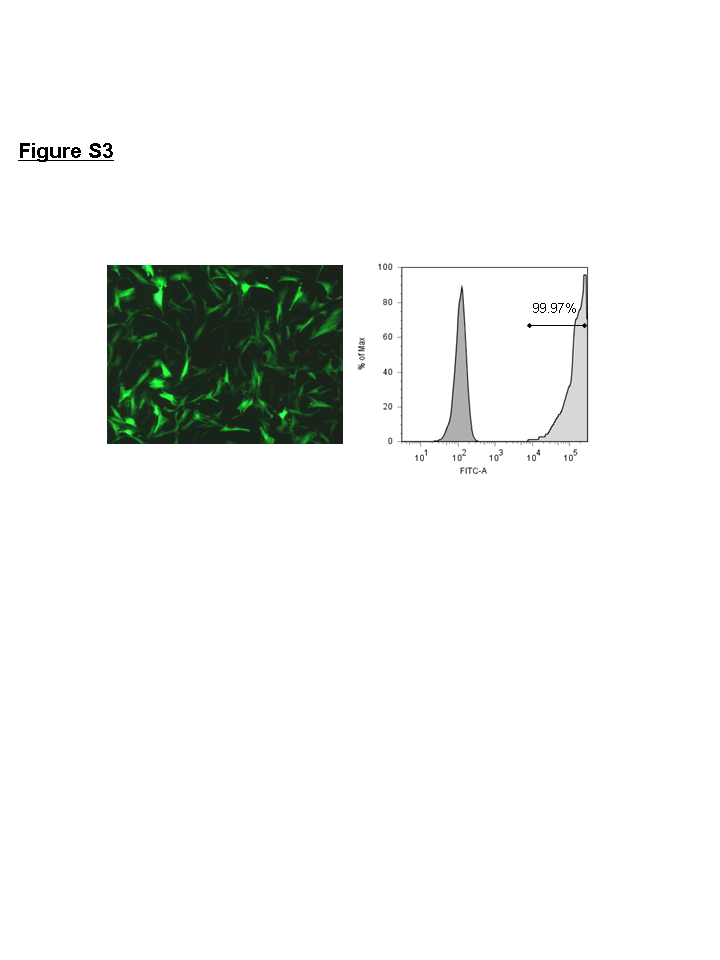

Supplement: Figure S3 — Transduction efficiency of hMSC by lentiviral vector was assessed with LV-CMV-GFP (MOI = 2). GFP expression was determined by microscopy and FACS. Untransduced MSC were used as negative (light grey) (C). (TIF) [file pone.0048093.s003.tif]
